# Keeping Places

**Published:** 1871-10

**Authors:** 


### KEEPING PLACES.

THERE are thousands of young men and women seeking for employment in city and country. On inquiry it will be found that they have had places, but have left them; and, many times, before they can get another situation their money is expended, and then the temptation is strong to fall into evil practices, which too often end in disease, disgrace, and crime. It rarely happens that a person is discharged from employment without cause, for employers are averse to change, and often keep persons who have glaring faults and defects rather than risk getting worse ones. It often happens that worthy and exemplary persons are dismissed for shortcomings of which they are entirely unconscious, and only need their attention to be called to the subject, to have them promptly rectified. To that vast multitude of persons who work for others, the following suggestions are made: —

1. Whatever you attempt, let it be done in the very best manner possible, and each subsequent time try to do it better than before.

2. Be at more pains to do a thing well than to do it quickly.

3. When you are asked to do a thing, do it cheerfully and promptly.

4. Do whatever is required of you, whether it is in the line of your particular duty or not.

5. Act always in the interest of your employer as far as is right.

6. Do what you see is wanting to be done, without waiting to be asked to do it.

7. Never say "I forgot it;" that is insult added to injustice, for it really means that you "did not care."

8. Never stop when you have done a thing which you were told to do, if by doing more, you can do it more perfectly.

9. Never refuse to lend a helping hand in emergencies.

10. Aim to do for your employer as well as you would do for yourself, in all things.

A strong reason for attending to these suggestions is, that your employer pays you your wages to the very last cent, and in whatever small respect you fail of your duty to him, you fail of compensation to him for the money paid you.

